# Impact of Guideline-Directed Medical Therapy on Left Ventricular Function in Heart Failure Patients with Conventional Right Ventricular Pacing

**DOI:** 10.3390/jcm15166452

**Published:** 2026-08-20

**Authors:** Liviu Cirin, Oana Pătru, Silvia Luca, Paul Ciubotaru, Roxana Buzaș, Constantin-Tudor Luca, Daniel Lighezan, Dragoș Cozma

**Affiliations:** 1 Doctoral School, “Victor Babes” University of Medicine and Pharmacy, 2 Eftimie Murgu Sq., 300041 Timisoara, Romania; cirin.liviu@umft.ro (L.C.); silvia.luca@umft.ro (S.L.); 2 Department of Internal Medicine I, “Victor Babes” University of Medicine and Pharmacy, 2 Eftimie Murgu Sq., 300041 Timisoara, Romaniabuzas.dana@umft.ro (R.B.); dlighezan@umft.ro (D.L.); 3 Department of Cardiology, “Victor Babes” University of Medicine and Pharmacy, 2 Eftimie Murgu Sq., 300041 Timisoara, Romania; constantin.luca@umft.ro (C.-T.L.); dragos.cozma@umft.ro (D.C.); 4 Institute of Cardiovascular Diseases Timisoara, 13A Gheorghe Adam Street, 300310 Timisoara, Romania; 5 Research Center of the Institute of Cardiovascular Diseases Timisoara, 13A Gheorghe Adam Street, 300310 Timisoara, Romania; 6 Center for Advanced Research in Cardiovascular Pathology and Hemostaseology, “Victor Babes” University of Medicine and Pharmacy, 2 Eftimie Murgu Sq., 300041 Timisoara, Romania

**Keywords:** pacing-induced cardiomyopathy, right ventricular pacing, heart failure, sacubitril/valsartan, sodium–glucose cotransporter-2 inhibitors, guideline-directed medical therapy, reverse remodeling, MAPSE

## Abstract

**Background/Objectives**: Heart failure (HF) in patients with conventional right ventricular pacing with significant pacing percentages is still a subject of concern, and management of HF in this population has historically been difficult; however, novel HF pillar medications, such as SGLT2 inhibitors (SGLT2i) and angiotensin receptor/neprilysin inhibitors (ARNi), have fundamentally transformed contemporary pharmacological HF management. The aim of this study was to assess left ventricular (LV) function after current guideline-directed medical therapy (GDMT) in patients with chronic right ventricular (RV) pacing and HF. **Methods**: Patients with a lifetime ventricular pacing percentage > 20% (Vp > 20%) and HF diagnosed according to ESC guideline criteria were included. Device interrogation and transthoracic echocardiography (TTE), including assessment of left ventricular ejection fraction (LVEF) and mitral annular plane systolic excursion (MAPSE), were performed at GDMT initiation and during subsequent follow-up. Changes in LVEF and MAPSE were assessed overall and according to baseline HF phenotype. Multivariable linear regression analyses were performed to identify independent predictors of changes in LVEF and MAPSE. **Results**: Among 550 conventionally paced patients screened for HF, 127 (23.1%) met the inclusion criteria and underwent GDMT initiation. Mean age was 68.2 ± 12.1 years, and mean follow-up duration was 10.2 ± 6.3 months. Baseline LVEF and MAPSE were 48.7 ± 6.3% and 12.0 ± 2.4 mm, respectively. All patients received SGLT2i therapy, while 24 (18.9%) received ARNi. Overall, LVEF increased by 1.94 ± 2.47% and MAPSE by 0.84 ± 0.96 mm (both *p* < 0.001). Patients with HFrEF (*n* = 25) showed an increase in LVEF of 5.84 ± 2.53% and MAPSE of 1.72 ± 1.21 mm. In patients with HFmrEF, LVEF increased by 2.50 ± 1.54% and MAPSE by 0.83 ± 0.92 mm, whereas in HFpEF, LVEF increased by 0.67 ± 0.78% and MAPSE by 0.58 ± 0.71 mm. NYHA functional class improved by at least one class in 100 patients (78.7%). In multivariable analysis, ARNi use was independently associated with greater improvement in LVEF and MAPSE, while lower baseline LV function was associated with greater subsequent improvement. **Conclusions**: In patients with HF and chronic RV pacing, GDMT was associated with modest but statistically significant improvements in LV systolic function, assessed by LVEF and MAPSE, across the HF spectrum. The greatest improvements were observed in patients with HFrEF. However, given the observational design, absence of a control group, and universal use of SGLT2i, these findings do not establish a causal treatment effect or demonstrate superiority of any specific GDMT combination. Prospective controlled studies are warranted to determine the clinical significance of these changes and the independent contribution of individual therapies.

## 1. Introduction

Conventional endocardial right ventricular (RV) antibradycardia pacing has been the mainstay of treatment for decades and is a well-documented technique. Historically, since the advent of pacemaker implants, the RV apex (RVA) has been a preferred site for pacing lead placement for multiple reasons, such as procedural ease of access and long-term lead stability. However, chronic RVA pacing has been shown to have deleterious effects on left ventricular (LV) function, being linked to an increased risk of heart failure (HF) and death. RVA pacing was widely performed and still is an integral part of bradyarrhythmia treatment for a significant proportion of patients receiving pacemakers. It is, however, proven to be a nonphysiological substitute for intrinsic ventricular activation over the His–Purkinje system and is a detrimental form of pacing in certain patient categories, increasing the risk of HF, particularly in patients with already abnormal LV function. Understanding this pathophysiology has driven the development of pacing technology to reduce the need for right ventricular apical pacing and the search for improved methods of ventricular pacing [1]. Conventional RV pacing (typically via an apically positioned RV lead) usually generates an iatrogenic left bundle branch block pattern on the surface ECG, causing asynchronous ventricular contraction. Evidence accumulated over the past decades highlights that right ventricular septal (RVS) pacing, or non-apical pacing, offers a more physiological activation pattern that mitigates some of the adverse effects associated with RVA [2]. Over time, this desynchrony may lead to a condition labeled as pacing-induced cardiomyopathy (PiCM)—a form of ventricular remodeling and systolic dysfunction due to cardiac pacing. PiCM is characterized by a decline in LVEF and HF symptoms in patients with high RV pacing burden, and clinical studies estimate that PiCM occurs in roughly 10–20% of pacemaker-dependent patients, especially when the RV pacing percentage is high. Risk appears to increase when over 20–40% of beats are RV-paced. Pathologically, RV pacing-induced HF shares many features with other forms of cardiomyopathy, such as RAAS activation, sympathetic upregulation, inflammation, and fibrosis, which contribute to disease progression [3,4,5,6,7]. Management traditionally centers on restoring synchrony by upgrading to physiological pacing via either traditional biventricular pacing (CRT) or conduction system pacing (His bundle or left bundle branch area pacing); however, the introduction og guideline-directed medical therapy (GDMT) for HF is also crucial. Among GDMT, the newer angiotensin receptor–neprilysin inhibitors (ARNi) and sodium–glucose cotransporter-2 inhibitors (SGLT2i) have emerged as key therapies in HF regardless of its origin. Sacubitril/valsartan and SGLT2i (e.g., dapagliflozin, empagliflozin) have in recent years become standard for heart failure according to both ESC and AHA/ACC/HFSA guidelines, in addition to the older mineralocorticoid receptor antagonists (MRAs) and beta-blockers (BB) [8,9]. No trial has been designed specifically to examine these agents in the pacing-induced HF phenotype. This observational study therefore aimed to characterize changes in echocardiographic LV function following the initiation of GDMT with SGLT2i and/or ARNi, along with MRAs and BB, in a cohort of conventionally RV-paced HF patients, contributing hypothesis-generating evidence to a clinically relevant but underexplored area.

## 2. Materials and Methods

### 2.1. Study Design and Population

This was a prospective, single-center, observational cohort study, which included 550 consecutive patients conventionally paced either from the RVA or from the RVS, with lifetime ventricular pacing percentages over 20% (Vp > 20%), who were screened for HF and diagnosed according to current ESC guidelines, including clinical assessment, echocardiographic findings, and elevated NT-proBNP levels. The final study group included 127 (23.09%) patients with HF and a high RV pacing burden with an indication for GDMT introduction between 2024 and 2026. The mean age of the group was 68.2 ± 12.1 years, while the mean follow-up period was 10.2 ± 6.3 months. We categorized patients into groups based on LVEF at inclusion as HFpEF (≥50%), HFmrEF (41–49%), and HFrEF (≤40%), according to the ESC guidelines. Patients in the HFpEF category received SGLT2i, MRA, and ARBs or ACEi, while the other 2 groups received all pillar medications, when possible, with ARNi doses titrated according to blood pressure tolerance. Exclusion criteria included patients with a GFR under 30 mL/min/1.72 m^2^, conduction system pacing leads, ICDs, patients with LVEF ≤ 35% who benefited from an upgrade to a form of cardiac physiologic pacing (CPP) such as biventricular CRT or conduction system pacing, and patients with chronic coronary syndrome and significant coronary artery disease (CAD) or other overt causes of HF (e.g., myocarditis, valvular heart disease, genetic cardiomyopathies, infiltrative diseases, substance-induced cardiomyopathy, and chemotherapy-induced cardiotoxicity) [10,11]. Device upgrades (to CRT or CSP) were considered for patients who exhibited refractory symptoms despite maximal GDMT and maintained a pacing burden >40%, typically evaluated at least 6 months after the initial evaluation. The study followed the Declaration of Helsinki guidelines and received approval from the local hospital ethics committee. All patients participating in the study signed informed consent beforehand. Device interrogation and TTE with LVEF% and MAPSE (mm), due to their validation in this subset of patients, ease of use, and reproducibility [12], were performed at initiation of GDMT and during subsequent follow-ups, among other parameters. The primary objective was to assess changes in LVEF, MAPSE, and NYHA class improvement after SGLT2i and/or ARNi introduction. Patients received GDMT according to contemporary recommendations, with BB therapy titrated individually to avoid unnecessary ventricular pacing, MRAs adjusted or initiated, spironolactone (25–50 mg OD), and loop diuretics (furosemide p.o.) adjusted according to congestion/volume status. Congestion and volume status were assessed using a multiparametric clinical approach, combining physical examination (auscultation, jugular venous pressure, peripheral edema) with point-of-care ultrasound (inferior vena cava diameter and lung ultrasound for B-lines). ACEis and ARBs (if previously used) were replaced with ARNi when and if possible in HFrEF and HFmrEF patients, and initiated if lacking in HFpEF. SGLT2i therapy was initiated with either dapagliflozin or empagliflozin (10 mg OD) in the absence of active UTIs or diabetic ketoacidosis (DKA). Sacubitril/valsartan was initiated at baseline with an initial dose (24/26 mg or 49/51 mg BID) according to blood pressure values, GFR, and serum potassium levels. Dose uptitration of ARNi to a maximum of 97/103 mg BID, if tolerable, was performed during outpatient follow-up visits at 4-to-8-week intervals.

### 2.2. Device Interrogation

**Figure 1:**
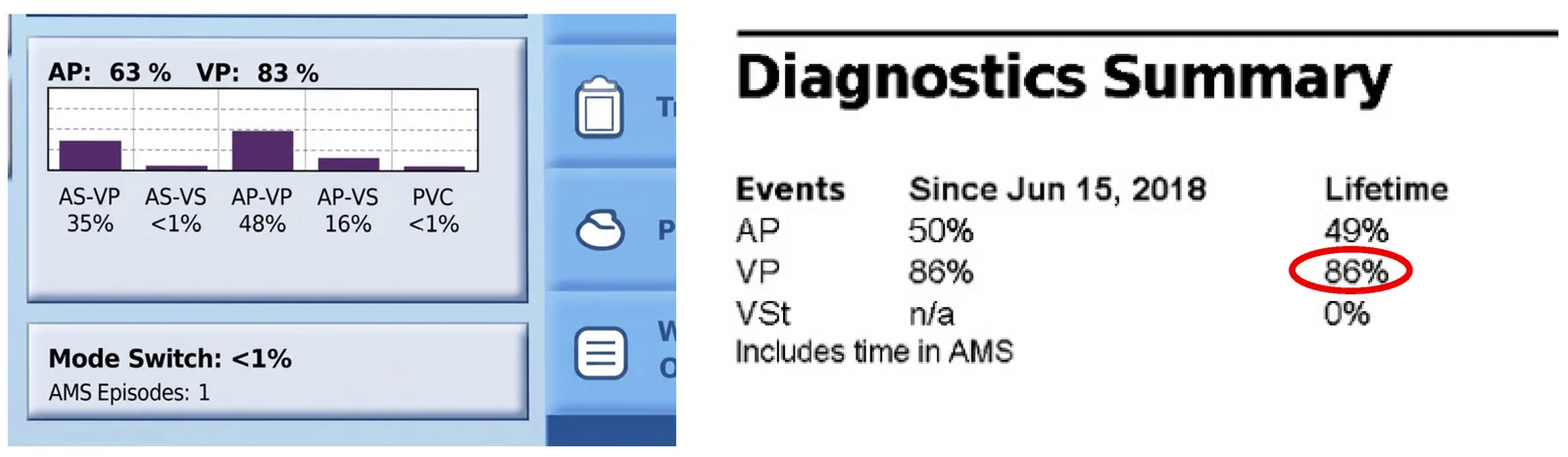
Example of pacemaker interrogation screen showing lifetime Vp percentage.

Standard device interrogation was performed initially to screen for a lifetime Vp > 20% and at follow-ups for patients with an HF diagnosis, as seen in Figure 1. Pacing and sensing parameters were noted, and device optimizations were performed as needed for each patient’s specific needs.

### 2.3. Transthoracic Echocardiographic Assessment

TTE examination and image analysis were performed by experienced, independent, blinded observers using a Vivid 9 ultrasound system from General Electric (Milwaukee, WI, USA) and a Philips Affiniti CVx platform (Koninklijke Philips N.V., Amsterdam, The Netherlands). TTE examinations were performed at diagnosis and initiation of GDMT and at subsequent follow-ups. All images were digitally stored and later analyzed using EchoPac software (version 203, General Electric Vingmed Ultrasound, Horten, Norway) and Ultrasound Workspace (version 3.0, Koninklijke Philips N.V., Amsterdam, The Netherlands). Echocardiographic image acquisition was performed while the patient was in the left lateral decubitus position, synchronized with the electrocardiogram. Apical four-chamber, two-chamber, and parasternal long- and short-axis views were obtained at a frame rate exceeding 40 frames per second (fps), covering at least 3 consecutive cardiac cycles. LVEF was measured using biplane Simpson’s method, while MAPSE was measured in the apical four-chamber view (A4C). The following parameters were also evaluated as part of the standard protocol regarding LV systolic, diastolic, and RV function: TDI s′ (cm/s) and e′ (cm/s), E/A ratio, E/e′ ratio, TAPSE (mm), and valvular pathology grades. Echocardiographic measurements were assessed in accordance with the guidelines of the European Association of Cardiovascular Imaging and the American Society of Echocardiography [13].

### 2.4. Lead Position

Apical or septal lead position was defined using 12-lead ECG paced QRS morphology and fluoroscopic confirmation. RVA pacing produces a wide LBBB pattern with negative QRS complexes in leads DII and DIII, while septal pacing tends to generate slightly narrower QRS complexes, which are positive in leads DII and DIII [14].

### 2.5. Statistical Analysis

Continuous variables were expressed as mean ± standard deviation (SD), and categorical variables were presented as absolute frequencies and percentages. The distribution of continuous variables was evaluated for normality using the Shapiro–Wilk test. Because the differences between baseline and follow-up echocardiographic parameters were not normally distributed, the non-parametric Wilcoxon signed-rank test was utilized to compare paired pre-treatment and post-treatment values of LVEF and MAPSE. Exploratory subgroup analyses were also performed by stratifying patients according to standard baseline LVEF categories. A multivariable linear regression analysis was employed to isolate the independent effect of specific pharmacological interventions on echocardiographic changes (ΔLVEF and ΔMAPSE). The multivariable model adjusted for age, male gender, baseline LVEF/MAPSE, pacing burden (Vp%), septal lead position, and specific GDMT classes (ARNi, ACEi/ARB, BB, MRA). Because SGLT2i therapy was universally prescribed across the cohort, it could not be evaluated as an independent variable. Consequently, the linear regression models assessed the additive, independent effects of ARNi, ACEi/ARBs, BB, and MRAs on top of baseline SGLT2i therapy. Statistical analysis was performed using IBM SPSS software (version 29.0.2.0) and MedCalc (version 22.021), and *p*-values < 0.05 were considered statistically significant.

## 3. Results

Among 550 conventionally paced patients screened for HF, 127 (23.1%) met the inclusion criteria and underwent GDMT initiation. The mean age was 68.2 ± 12.1 years, 70 (55.1%) were male, and the mean follow-up duration was 10.2 ± 6.3 months. Baseline LVEF and MAPSE were 48.7 ± 6.3% and 12.0 ± 2.4 mm, respectively. Ninety-five patients (74.8%) underwent RVA pacing, whereas 32 (25.2%) underwent RVS pacing. Baseline patient characteristics are summarized in Table 1, and GDMT distribution is presented in Table 2.

Introduction of GDMT was associated with significant improvements in both LVEF and MAPSE across the entire cohort (all *p* < 0.001) (Table 3). The greatest absolute improvement was observed in patients with HFrEF, in whom mean LVEF increased by 5.84 ± 2.53% and MAPSE by 1.72 ± 1.21 mm (both *p* < 0.001). Patients with HFmrEF also demonstrated significant improvement (ΔLVEF +2.50 ± 1.54%; ΔMAPSE +0.83 ± 0.92 mm), whereas patients with HFpEF showed smaller but still statistically significant changes (ΔLVEF +0.67 ± 0.78%; ΔMAPSE +0.58 ± 0.71 mm). In parallel, E/e′ significantly decreased following GDMT initiation, from 16.2 ± 6.2 at baseline to 14.9 ± 6.19 at follow-up (ΔE/e′ −1.3, *p* < 0.001). The greatest reduction was observed in patients with HFrEF (ΔE/e′ −2.3, from 16.5 ± 6.0 to 14.2 ± 6.0, *p* < 0.001), followed by HFmrEF (ΔE/e′ −1.5, from 14.8 ± 5.5 to 13.3 ± 5.4, *p* = 0.0055) and HFpEF (ΔE/e′ −1.0, from 17.2 ± 6.5 to 16.2 ± 6.3, *p* < 0.001).

Improvement in longitudinal systolic function, assessed by MAPSE, paralleled the improvement in global systolic function after GDMT optimization. NYHA functional class improved in 100 patients (78.7%), remained unchanged in 25 (19.6%), and worsened in only two patients (1.7%).

**Figure 2:**
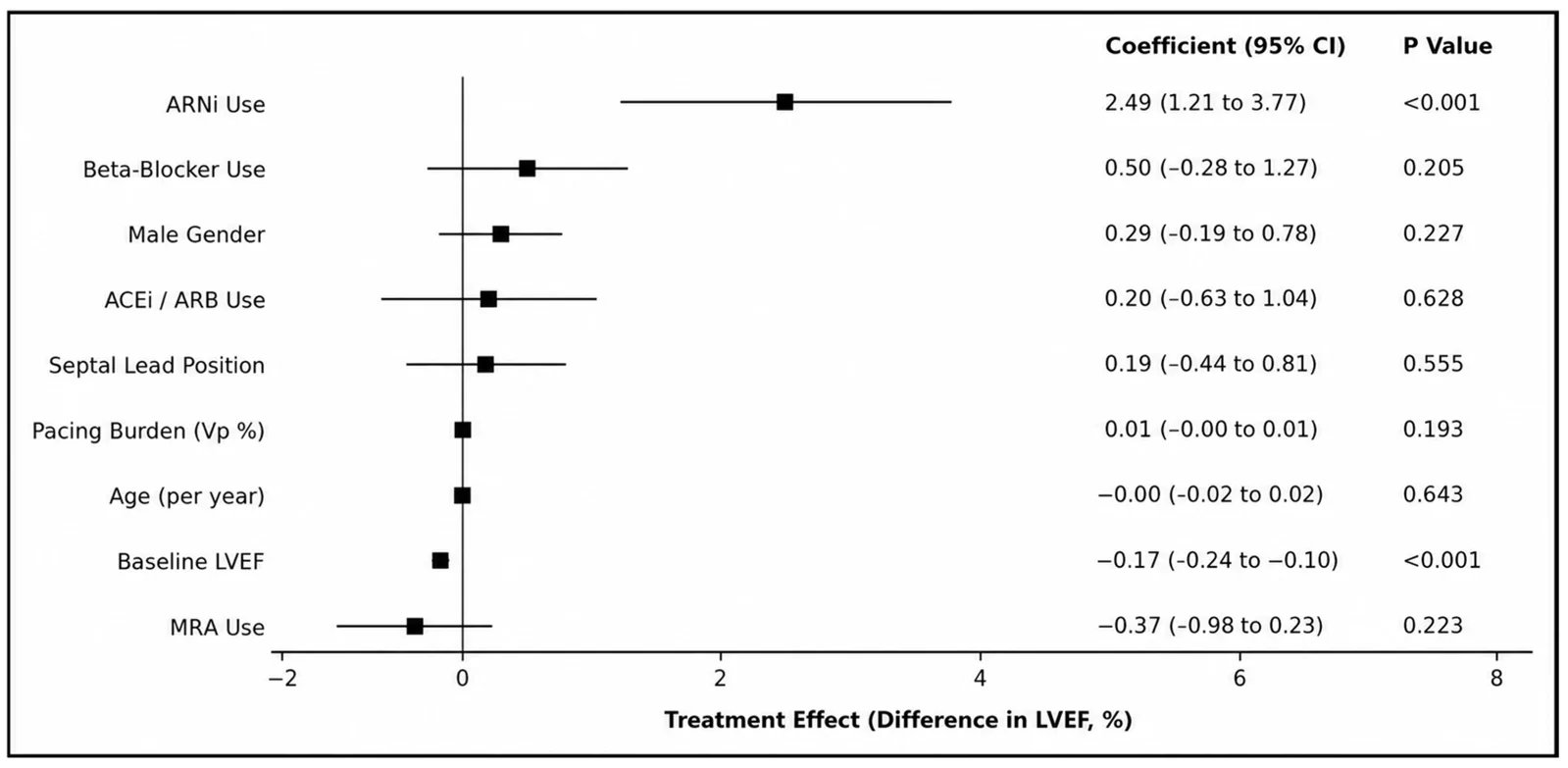
Multivariable linear regression analysis showing independent predictors of change in LVEF (ΔLVEF). Regression coefficients are presented with 95% CI. Positive coefficients indicate greater improvement in LVEF following GDMT initiation. ARNi use was the only independent pharmacological predictor of LVEF improvement, whereas baseline LVEF was inversely associated with treatment response.

Because SGLT2 inhibitor therapy was prescribed in all patients, multivariable linear regression was performed to evaluate the independent contribution of the remaining GDMT components together with relevant clinical covariates (Figure 2). After adjustment for age, sex, pacing burden, septal lead position, and baseline LVEF, ARNi use remained the only pharmacological variable independently associated with greater improvement in LVEF (*β* = 2.49, 95% CI 1.21–3.77, *p* < 0.001). Baseline LVEF was inversely associated with subsequent LVEF improvement (*β* = −0.17, 95% CI −0.24 to −0.10, *p* < 0.001), indicating greater recovery among patients with lower baseline systolic function. No independent associations were observed for beta-blocker therapy, MRA therapy, ACEi/ARB therapy, pacing burden, septal lead position, age, or sex (all *p* > 0.05).

**Figure 3:**
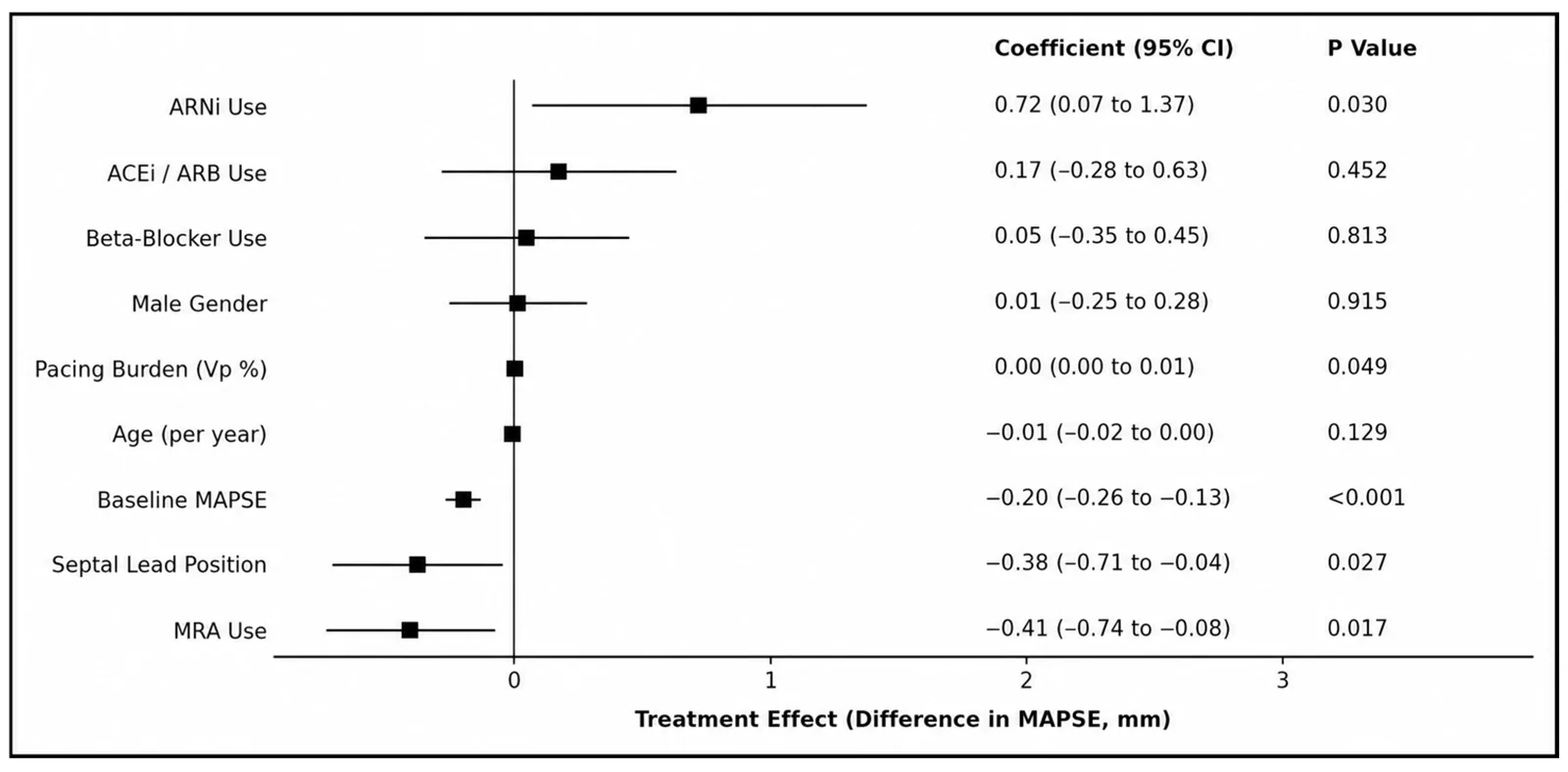
Multivariable linear regression analysis showing independent predictors of change in MAPSE (ΔMAPSE). Regression coefficients are presented with 95% CI. Positive coefficients indicate greater improvement in MAPSE following GDMT initiation.

A separate multivariable linear regression model was constructed to identify independent predictors of longitudinal LV functional recovery, assessed by changes in MAPSE (Figure 3). ARNi use remained independently associated with greater improvement in MAPSE (*β* = 0.72, 95% CI 0.07–1.37, *p* = 0.030). Lower baseline MAPSE independently predicted greater subsequent improvement (*β* = −0.20, 95% CI −0.26 to −0.13, *p* < 0.001). In addition, septal lead position (*β* = −0.38, 95% CI −0.71 to −0.04, *p* = 0.027), MRA therapy (*β* = −0.41, 95% CI −0.74 to −0.08, *p* = 0.017) and pacing burden (*β* = 0.00, 95% CI 0.00–0.01, *p* = 0.049) were independently associated with changes in MAPSE, whereas ACEi/ARB therapy, BB therapy, age, and sex were not significant predictors.

**Figure 4:**
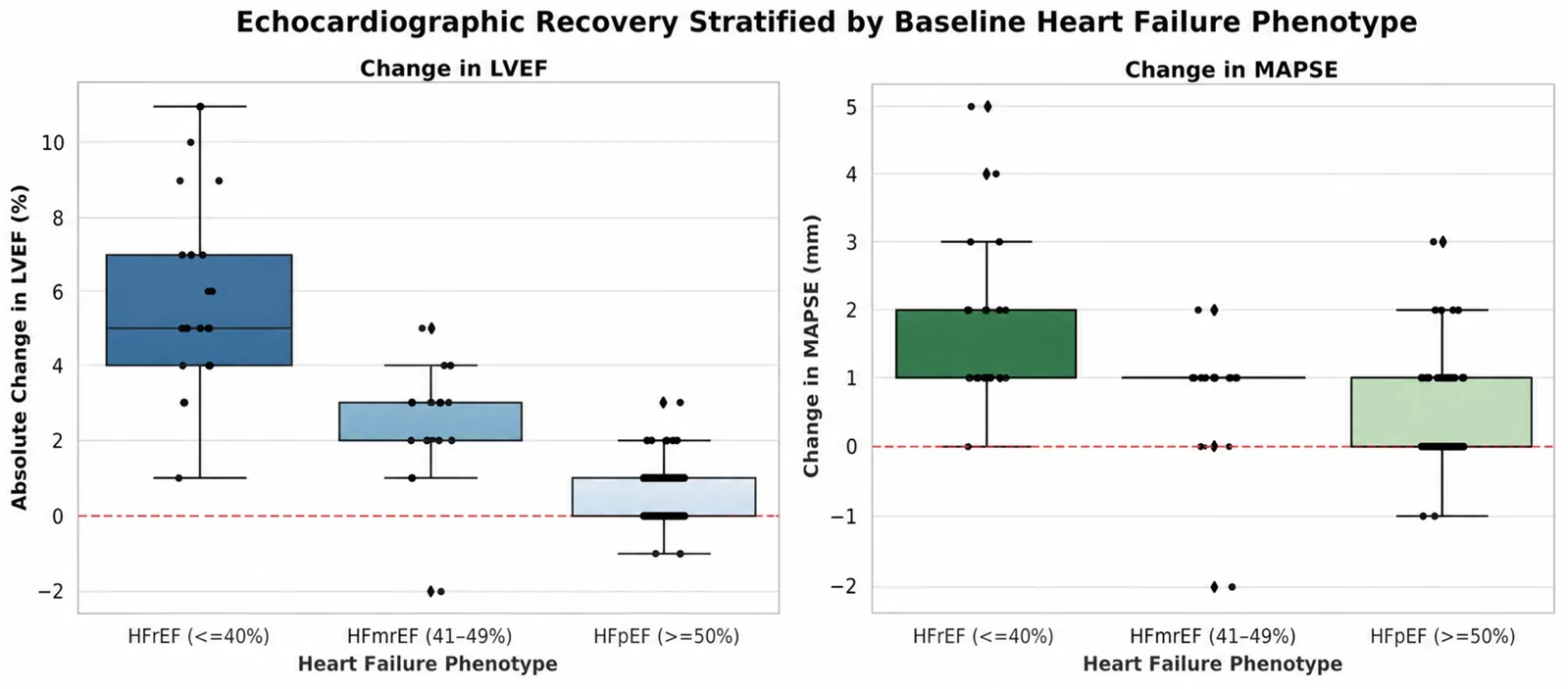
Echocardiographic changes stratified by baseline HF phenotype; dual-panel boxplots illustrating ΔLVEF (**left panel**) ΔMAPSE (**right panel**) from baseline to follow-up across three heart failure phenotypes; the overlaid black dots represent individual patient values (stripplot), demonstrating the distribution and density of the data. Diamond symbols indicate outlier observations beyond the boxplot whiskers (i.e., values more than 1.5 × IQR from the first or third quartile). The dashed red line at 0 indicates no change from baseline.

Figure 4 illustrates the distribution of individual changes in LVEF and MAPSE from baseline to follow-up across the three HF phenotypes. Patients with HFrEF showed the greatest magnitude and variability of improvement in both LVEF and MAPSE, whereas patients with HFpEF showed smaller changes, with HFmrEF demonstrating an intermediate pattern.

## 4. Discussion

### 4.1. Device Upgrade to CSP and Pharmacological Therapy

Although pacing sites such as the interventricular septum or right ventricular outflow tract have been explored in conventional pacing, most studies regarding outcomes in these cases have been small, and the benefit in terms of HF/PiCM prevention remains largely unproven or, at the very least, controversial [14]. Upgrading to biventricular pacing (BiV-CRT) is the standard viable option for minimizing pacing-induced HF and is formally established in current guidelines; however, procedural success is not always guaranteed, the procedure carries a significant rate of potential complications, and studies have emphasized the not insignificant rate of nonresponders to CRT (~30%) [15]. Conduction system pacing (CSP) is a more recent option, with promising evidence; however, its availability and operator experience (with a significant procedural learning curve) can be a burden for smaller implant centers. Also worth mentioning is that current evidence only provides a class IIa/b indication according to the 2021 ESC Guidelines on cardiac pacing and cardiac resynchronization therapy [16]. Thus, there is a clear need to explore the potential benefit of pharmacological treatment in these patients. The findings suggest that prompt GDMT initiation in patients awaiting or anticipating device optimization is appropriate and may partially attenuate adverse LV remodeling in the interim. For patients in whom CRT or CSP is technically feasible and clinically indicated, device intervention remains the priority, with GDMT serving a synergistic, not substitutive, role.

### 4.2. Pharmacotherapy

Although no trial has exclusively studied pacemaker recipients receiving ARNi, robust evidence from HFrEF trials and case series supports ARNi use in this context. Sacubitril/valsartan was shown to be superior to ACE inhibition in the PARADIGM-HF trial (which also enrolled patients with ICDs who likely also had high pacing needs) [17]. In PARADIGM-HF, sacubitril/valsartan reduced the composite of cardiovascular death or HF hospitalization by ~20% compared to enalapril (21.8% vs. 26.5% event rate over ~2.25 years, HR 0.80, *p* < 0.001) [17]. Importantly, ARNi therapy leads to reverse remodeling of the left ventricle; multiple studies (e.g., PROVE-HF) have documented significant LVEF improvement and LV volume reduction after initiation of sacubitril/valsartan. This reverse remodeling was seen even in those with prior “non-ischemic” cardiomyopathy aetiologies, a category that would include PiCM [18]. Observational evidence in pacemaker patients aligns with trial data. Case reports and series of PiCM patients show that introducing sacubitril/valsartan (in addition to β-blockers and MRAs) often stabilizes or modestly improves LVEF, though in advanced cases device intervention is still needed. For example, one case of PiCM (LVEF dropped from 61% to 35% with >90% RV pacing) was managed for a year with optimized medical therapy including sacubitril/valsartan, a beta-blocker, and spironolactone. While the patient’s EF remained low (~36%) until the pacing strategy changed, ARNi use was associated with symptom management and prevention of further decline [19]. These real-world experiences underscore that ARNi alone may not fully reverse severe dyssynchrony-induced dysfunction, but it likely blunts HF progression until mechanical synchrony can be restored [20]. Notably, ARNi benefits the right ventricle as well; recent data suggest that sacubitril/valsartan can improve RV function (e.g., reducing RV volumes, improving TAPSE and RV strain) in non-ischemic cardiomyopathies [21]. This could be relevant because chronic RV pacing can also impair RV performance (due to interventricular interdependence and possibly pacing-induced tricuspid regurgitation) [22].

SGLT2 inhibitors have rapidly moved to frontline therapy in HF across the EF spectrum. Their relevance for RV-paced patients lies in their ability to improve cardiac outcomes and function regardless of HF etiology. In the landmark DAPA-HF trial [23], dapagliflozin significantly reduced the risk of worsening HF or cardiovascular death (the primary composite outcome) by about 26% relative to placebo (HR ~0.74). This corresponded to a 4.9% absolute risk reduction in CV death or HF hospitalization over 18 months (16.3% event rate on dapagliflozin vs. 21.2% on placebo). All-cause mortality was also lower (11.6% vs. 13.9%, a 2.3% absolute decrease) [23]. When combined, ARNi and SGLT2i may have additive effects: a recent prospective study of 100 HF patients on sacubitril/valsartan found that adding an SGLT2 inhibitor led to greater improvements in LVEF and cardiac dimensions than ARNi alone. In three months, patients on ARNi + SGLT2i showed significant gains in LVEF and reductions in LV volumes; multivariable analysis confirmed a superior remodeling effect with dual therapy (*p* < 0.001 for LVEF benefit vs. ARNi alone). Notably, this study also documented improved TAPSE and RV S’ velocity with ARNi + SGLT2i, indicating enhanced right ventricular function. For a patient with PICM, this suggests that combination therapy can facilitate biventricular reverse remodeling, partly countering the dyssynchrony-induced changes [24]. In clinical case series of PICM, however, medication alone did not always provide satisfactory LVEF improvement, reinforcing that addressing the electrical dyssynchrony (through CRT or CSP) is required for significant improvement [25,26,27].

While the improvements in LVEF, MAPSE, and E/e′ reached statistical significance, the absolute magnitude of these changes was modest. When contextualizing these findings against major HF trials (such as PARADIGM-HF, DAPA-HF, and EMPEROR-Reduced) [17,23], the observed improvement in LVEF in our study appears blunted. We hypothesize that the persistent electrical and mechanical dyssynchrony imposed by chronic high-burden RV pacing acts as a constant opposing force against the reverse remodeling effects of GDMT. Nevertheless, in a cohort subjected to continuous dyssynchrony, even modest structural recoveries can correlate with significant symptom stabilization and reduced filling pressures. This reinforces the importance of considering functional capacity and patient-centered outcomes alongside conventional echocardiographic measures when assessing response to contemporary HF therapy [28]. GDMT remains crucial, with one analysis showing that even after CRT, patients on sacubitril/valsartan continued to have fewer HF events than those on ACEi (i.e., the drug benefits persist alongside device therapy) [29]. No specific registry data yet quantify outcomes of ARNi/SGLT2i specifically in pacing-induced HF vs. non-paced HF, but given their mechanisms, there is no reason to expect a lesser benefit.

### 4.3. Ongoing Trials and Future Directions

In the specific context of conventional RV pacing-induced ventricular dysfunction, there are no pharmacotherapy trials dedicated solely to this subgroup of patients, largely because the emphasis in recent decades has been on device-based solutions. However, ongoing HF trials continue to broaden the indications of ARNi and SGLT2i, which will inevitably include patients with pacemakers. We have solid evidence from trials of ARNi (PARADIGM-HF 2014, PIONEER-HF 2019) and SGLT2i (DAPA-HF 2019, DELIVER 2022, EMPEROR-Reduced 2020, EMPEROR-Preserved 2021, EMPULSE 2022) benefits across the HF spectrum [17,23,29,30,31,32,33,34,35,36,37,38,39,40]. There is also interest in whether ARNi or SGLT2i have anti-arrhythmic benefits in patients with devices and ICDs. Some studies seem to suggest that sacubitril/valsartan reduces ventricular arrhythmias and sudden death compared to enalapril, and SGLT2i may reduce the incidence of atrial fibrillation and ventricular tachyarrhythmias in HF [17,23,29,30,31,32,33,34,35,36,37,38,39,40]. This is pertinent because patients with LVEF ≤ 35% often receive upgrades to ICDs (CRT-D) according to current ESC guidelines (IIa B) [16], and if ARNi/SGLT2i introduction lowers arrhythmia burden, that would be an added advantage.

At present, CRT upgrade remains the most effective treatment for established PiCM/HF, while pharmacologic therapy should be seen as complementary, improving hemodynamic and neurohormonal activation and outcomes during the period before and after a CRT upgrade. As of this publication, no specific “ARNi + SGLT2i vs. BiV-CRT” trial exists in such patient populations, and a case with clear HF attributed to RV pacing should benefit from both an upgrade to a BiV-CRT device and optimal, guideline-directed medical therapy. However, scenarios in which BiV-CRT or CSP are not immediately available or technically feasible are not uncommon, and in such cases no data have been published on the effect of optimal GDMT in such patients. Still, more evidence is required, underscoring that novel drugs often need to be paired with invasive pacing strategies, including reintervention for device upgrade. We concur that more data on ARNi in specific subgroups (e.g., heart failure in the context of conduction disease) are required, and if these drugs show arrhythmia-reducing properties, that could particularly benefit device patients. Registries have been collecting data on sacubitril/valsartan use in patients with ICDs to further characterize its benefits in those populations [41]. Modern pacemakers and ICDs can also measure intrathoracic impedance (congestion) and even estimate intracardiac pressures, data that should guide earlier diagnosis of HF and initiation of ARNi and/or SGLT2i if these early signs develop. Looking ahead, integrated management of patients with AVB or significant ventricular pacing needs will likely involve choosing a physiologic pacing technique to avoid dyssynchrony from the very start and treating any emerging LV dysfunction with the full spectrum of HF medication. Further studies are needed to clarify why some patients with high RV pacing burdens develop HF while others do not, and what the specific risk factors and outcome predictors are among these patients.

### 4.4. Study Limitations

Several limitations of our study need to be acknowledged, such as a relatively short follow-up period, the single-center design, lack of a control group, and the smaller number of HFrEF patients included, who usually benefit from device upgrades to traditional CRT or CSP. The absence of left ventricular global longitudinal strain (GLS) analysis is a notable limitation in thoroughly assessing myocardial deformation and risk stratification. Previous work has demonstrated that advanced echocardiographic parameters, including GLS and mechanical dispersion, may provide incremental prognostic information beyond conventional measures of LV systolic function, with E/e′ also incorporated into the assessment of cardiovascular risk in patients with HF [42]. Without a dedicated control group, we cannot definitively rule out the influence of the natural clinical course, dynamic changes in volume status, or regression to the mean. The study group is heavily dominated by HFpEF, with most patients receiving SGLT2i monotherapy. Because 100% of the patient cohort received SGLT2 inhibitors, it is statistically impossible to determine their independent contribution to the observed improvement in LVEF and MAPSE. Another potential limitation is the overwhelming use of passive fixation leads in our study group and an unbalanced number of RVA vs. RVS pacing cases, with septal pacing being predominant in the HFrEF group, possibly due to operator choice at the time of implantation. It is reasonable to assume that alternative pacing lead positions achievable by using active fixation leads might yield different long-term results.

Even though ESC guidelines [8] provide a class IIb indication for ARNi in HFmrEF, the national reimbursement scheme does not insure ARNi administration in this subset of patients, only the HFrEF category; thus, the number of patients on ARNi with mildly reduced LVEF in our study is negligible. The structural confounding introduced by policy restrictions limits direct subgroup comparisons and prevents reliable comparisons across subgroups. Finally, no data on HF hospitalization, mortality, or device upgrade rates were formally collected as endpoints in this trial, which constitutes a significant limitation.

### 4.5. Summary

In summary, the current pharmacological paradigm is to treat HF in RV-paced patients or PiCM as one would any other non-ischemic cardiomyopathy, initiating and uptitrating ARNi and SGLT2i (along with the rest of guideline-directed HF medication) promptly, unless otherwise contraindicated. This approach is supported by pathophysiologic reasoning and extrapolation from multiple clinical trials showing reverse remodeling, improved LVEF, reduced HF hospitalization, and mortality benefits with these drugs. Prospective controlled trials with hard clinical endpoints are needed to definitively characterize the role and potential synergy of ARNi and SGLT2i in this specific and clinically important patient population. While no drug for pacing-induced HF exists yet, the consistent message from our study and the literature in general is that ARNi and SGLT2i confer substantial cardiac benefit in this subset of patients with HF, alongside the rest of HF pillar medication.

## 5. Conclusions

Current HF pillar medication, including the more recent ARNi and SGLT2i, was associated with a significant increase in LVEF and MAPSE in conventionally RV-paced patients with HF across the spectrum over a mean follow-up of approximately 10 months. Patients with the most depreciated LV function (HFrEF) seem to have derived the greatest increase in LVEF and MAPSE, which might suggest that combined SGLT2i, ARNi, MRA, and BB therapy may provide added benefit over the introduction of individual therapies alone. Clinical improvement, as reflected by NYHA class, was also observed in the vast majority of patients. The data do not support inferring that pharmacological therapy can substitute for device-based resynchronization in patients with clear indications for CPP (CRT or CSP). Rather, they suggest that contemporary HF pharmacotherapy should be promptly initiated in conventionally RV-paced patients with HF, as a complement to, not in lieu of, device optimization when indicated. Early recognition of HF in this subgroup of HF patients is crucial for prompt initiation of contemporary HF pharmacological therapy, in concert with device optimization to obtain maximum benefit.

## Figures and Tables

**Table 1:** Baseline study population characteristics.

	All Patients *n* = 127	Patients with HFpEF *n* = 84	Patients with HFmrEF *n* = 18	Patients with HFrEF *n* = 25
Age, years (±SD)	68.2 ± 12.1	67 ± 13.1	70.7 ± 8.8	70.4 ± 9.9
Gender, male (%)	70 (55.1%)	41 (48.8%)	11 (61.1%)	16 (64.0%)
NTproBNP, pg/mL	2390 ± 2078	924 ± 611	1811 ± 923	4463 ± 2835
RVA pacing	95 (74.8%)	69 (82.1%)	15 (83.3%)	11 (44.0%)
LVEF, %	48.7 ± 6.3	52.4 ± 2.4	46.3 ± 1.0	37.8 ± 3.1
MAPSE, mm	12.0 ± 2.4	12.9 ± 1.9	12.0 ± 2.1	8.9 ± 1.4
TAPSE, mm	19.0 ± 5.3	21 ± 7.1	19 ± 4.3	17 ± 5.1
E/e′	16.2 ± 6.2	17.2 ± 6.5	14.8 ± 5.5	16.5 ± 6.0
Hypertension, %	72.3	83	70	64
Atrial fibrillation, %	34	45	35	22
Type 2 DM, %	33.3	36	33	31
Mean pacing burden (%)	62.65 ± 28.31	55.86 ± 29.01	72.56 ± 22.93	78.36 ± 20.81

**Table 2:** GDMT coverage across study cohort.

	All Patients *n* = 127	Patients with HFpEF *n* = 84	Patients with HFmrEF *n* = 18	Patients with HFrEF *n* = 25
ARNi, *n* (%)	24 (18.9%)	0 (0%)	2 (11.1%)	22 (88.0%)
SGLT2i, *n* (%)	127 (100%)	84 (100%)	18 (100%)	25 (100%)
Beta-blockers, *n* (%)	37 (29.1%)	4 (4.8%)	10 (55.6%)	23 (92.0%)
MRA, *n* (%)	98 (77.2%)	58 (69.0%)	17 (94.4%)	23 (92.0%)
ACEi/ARB, *n* (%)	90 (70.9%)	72 (85.7%)	15 (83.3%)	3 (12.0%)

**Table 3:** Echocardiographic outcomes after GDMT introduction.

	All Patients *n* = 127	HFrEF *n* = 25	HFmrEF *n =* 18	HfpEF *n* = 84
LVEF Outcomes (%)				
Follow-up LVEF (mean ± SD)	50.6 ± 4.5	43.6 ± 4.2	48.8 ± 1.9	53.0 ± 2.0
Absolute change (ΔLVEF)	+1.94 ± 2.47	+5.84 ± 2.53	+2.50 ± 1.54	+0.67 ± 0.78
*p*-value (vs. baseline)	<0.001	<0.001	<0.001	<0.001
MAPSE Outcomes (mm)				
Follow-up MAPSE (mean ± SD)	12.8 ± 2.0	10.6 ± 1.8	12.8 ± 1.8	13.4 ± 1.6
Absolute change (ΔMAPSE)	+0.84 ± 0.96	+1.72 ± 1.21	+0.83 ± 0.92	+0.58 ± 0.71
*p*-value (vs. baseline)	<0.001	<0.001	0.006	<0.001
E/e′ Outcomes				
Follow-up E/e′ (mean ± SD)	14.9 ± 6.19	14.2 ± 6.0	13.3 ± 5.4	16.2 ± 6.3
Absolute change (ΔE/e′)	−1.3	−2.3	−1.5	−1.0
*p*-value (vs. baseline)	<0.001	<0.001	0.0055	<0.001

## Data Availability

The original contributions presented in this study are included in the article. Further inquiries can be directed to the corresponding author.

## Abbreviations

The following abbreviations are used in this manuscript:

A4C	Apical Four-Chamber View
ACEi	Angiotensin-Converting Enzyme Inhibitor
ARB	Angiotensin II Receptor Blocker
ARNi	Angiotensin Receptor–Neprilysin Inhibitor
BB	Beta-Blocker
BiV-CRT	Biventricular Cardiac Resynchronization Therapy
CAD	Coronary Artery Disease
CI	Confidence Interval
CPP	Cardiac Physiologic Pacing
CRT	Cardiac Resynchronization Therapy
CRT-D	Cardiac Resynchronization Therapy with Defibrillator
CSP	Conduction System Pacing
DKA	Diabetic Ketoacidosis
ECG	Electrocardiogram
ESC	European Society of Cardiology
GDMT	Guideline-Directed Medical Therapy
GFR	Glomerular Filtration Rate
HF	Heart Failure
HFmrEF	Heart Failure with Mildly Reduced Ejection Fraction
HFpEF	Heart Failure with Preserved Ejection Fraction
HFrEF	Heart Failure with Reduced Ejection Fraction
ICD	Implantable Cardioverter-Defibrillator
LBBB	Left Bundle Branch Block
LV	Left Ventricular
LVEF	Left Ventricular Ejection Fraction
MAPSE	Mitral Annular Plane Systolic Excursion
MRA	Mineralocorticoid Receptor Antagonist
NT-proBNP	N-terminal Pro-B-type Natriuretic Peptide
NYHA	New York Heart Association
PiCM	Pacing-Induced Cardiomyopathy
QRS	QRS Complex
RAAS	Renin–Angiotensin–Aldosterone System
RV	Right Ventricular
RVA	Right Ventricular Apex
RVS	Right Ventricular Septal
SD	Standard Deviation
SGLT2i	Sodium–Glucose Cotransporter-2 Inhibitor
TAPSE	Tricuspid Annular Plane Systolic Excursion
TDI	Tissue Doppler Imaging
TTE	Transthoracic Echocardiography
UTI	Urinary Tract Infection
Vp	Ventricular Pacing Percentage
